# P-434. Role of Thalidomide in Children with Central Nervous System Tuberculosis

**DOI:** 10.1093/ofid/ofaf695.650

**Published:** 2026-01-11

**Authors:** Dhruv Gandhi, Minnie Bodhanwala, Ira Shah

**Affiliations:** Bai Jerbai Wadia Hospital for Children, Mumbai, India, West Monroe, LA; Wadia Group of Hospitals, Mumbai, India, Mumbai, Maharashtra, India; Bai Jerbai Wadia Hospital for Children, Mumbai, India, West Monroe, LA

## Abstract

**Background:**

Corticosteroids have long been used in the treatment of central nervous system tuberculosis (CNS-TB) to reduce local inflammation and the spread of the disease. However, in cases of non-response or paradoxical response to steroids or steroid-induced adverse effects, an alternative anti-inflammatory agent must be sought for. Thalidomide, an inhibitor of tumour necrosis factor-alpha, may play a role in reducing the disease severity in such patients. The aim of this study is to determine the role of thalidomide in the treatment of paediatric CNS-TB in a cohort of Indian children.

**Table 1:**
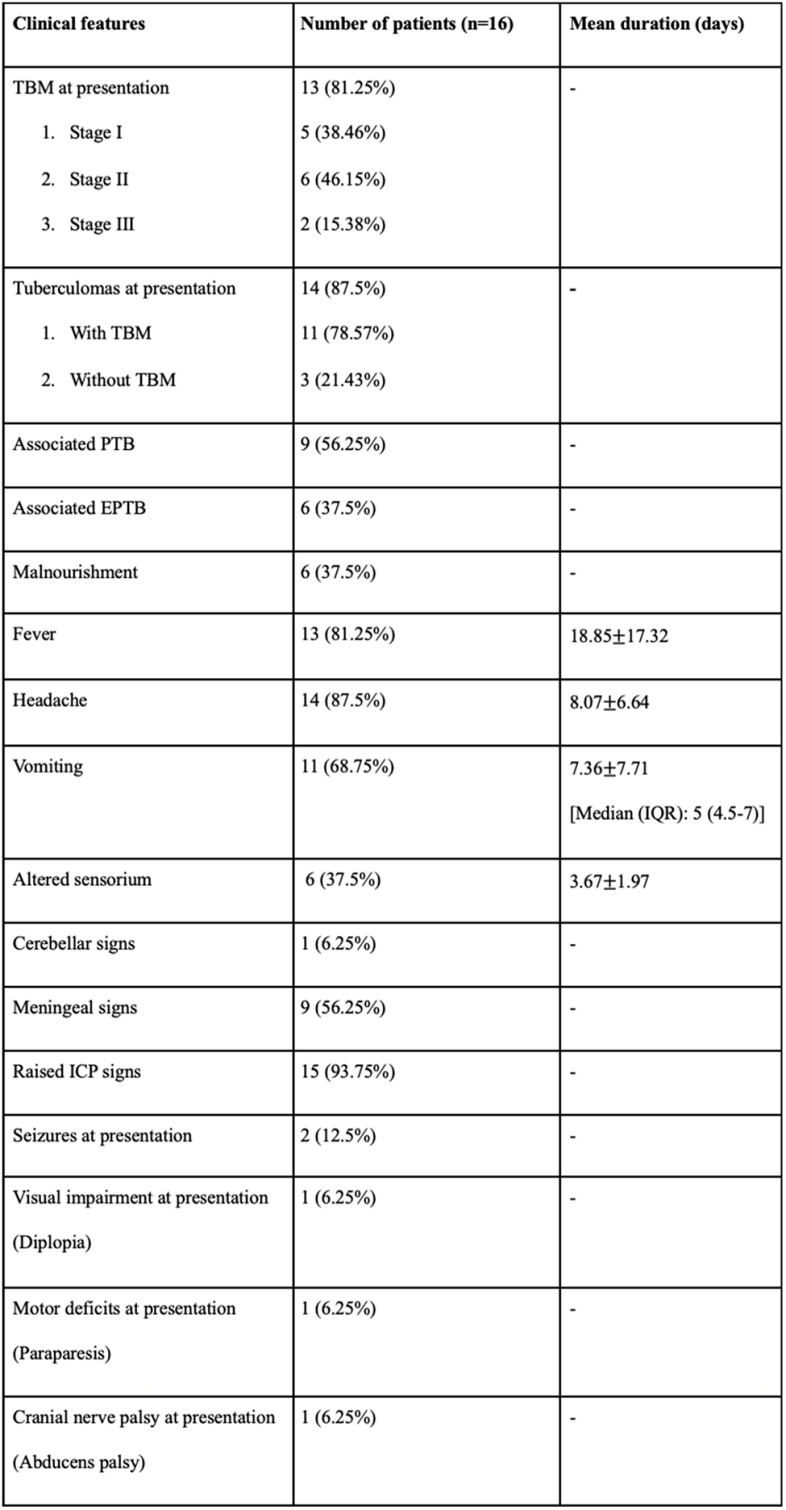
Clinical manifestations of the patients at presentation Note: TBM- tuberculous meningitis, PTB- Pulmonary tuberculosis, EPTB- extrapulmonary tuberculosis, IQR- interquartile range, ICP- intracranial pressure.

**Table 2:**
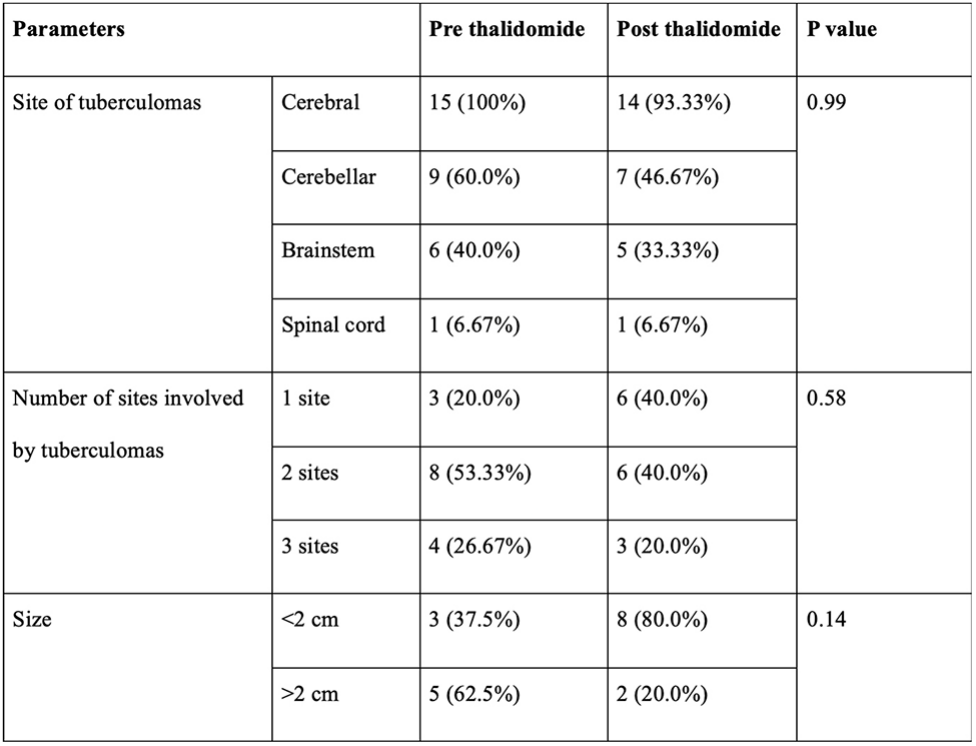
Radiological features of the patients before and after thalidomide exposure

**Methods:**

A retrospective single centre study was conducted from December 2018 to July 2024 and included 16 children less than 18 years of age, diagnosed with CNS-TB, having persistent tuberculomas in spite of steroid and anti-tubercular therapy (ATT), and having received thalidomide at 3 mg/kg/day. Radiological assessment for the number and size of tuberculomas pre- and post-thalidomide was done to determine the therapeutic response. Adverse reactions to steroids and thalidomide were also determined.

**Table 3:**
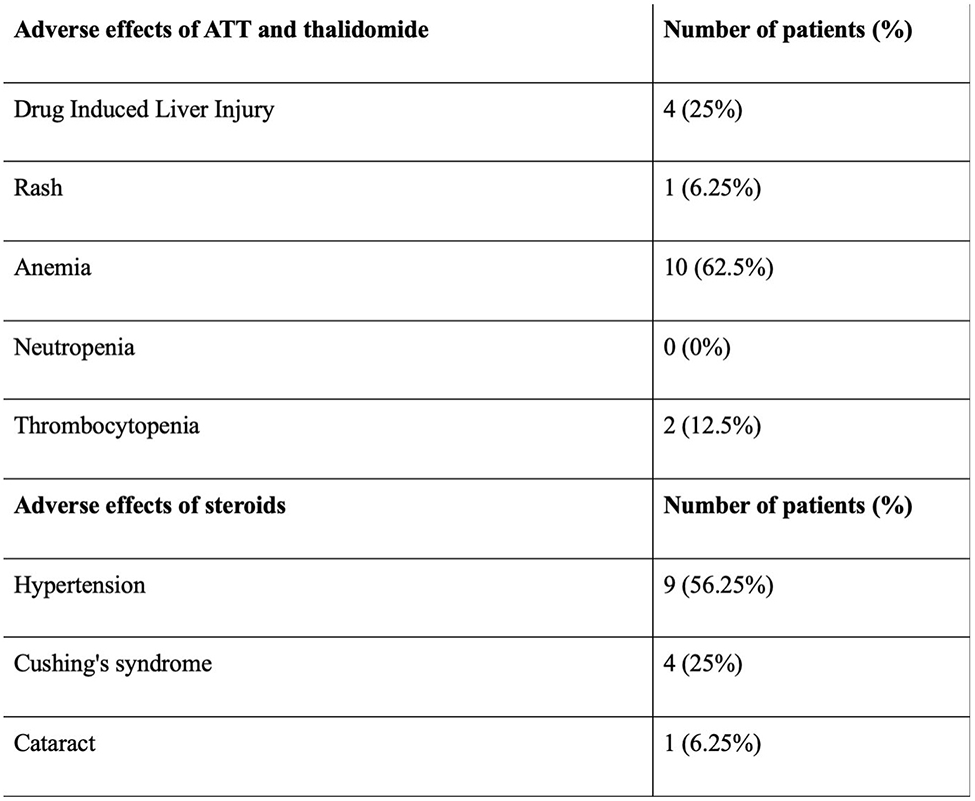
Adverse effects of anti-tubercular therapy, thalidomide and steroids Note: ATT- anti-tubercular therapy.

**Table 4:**
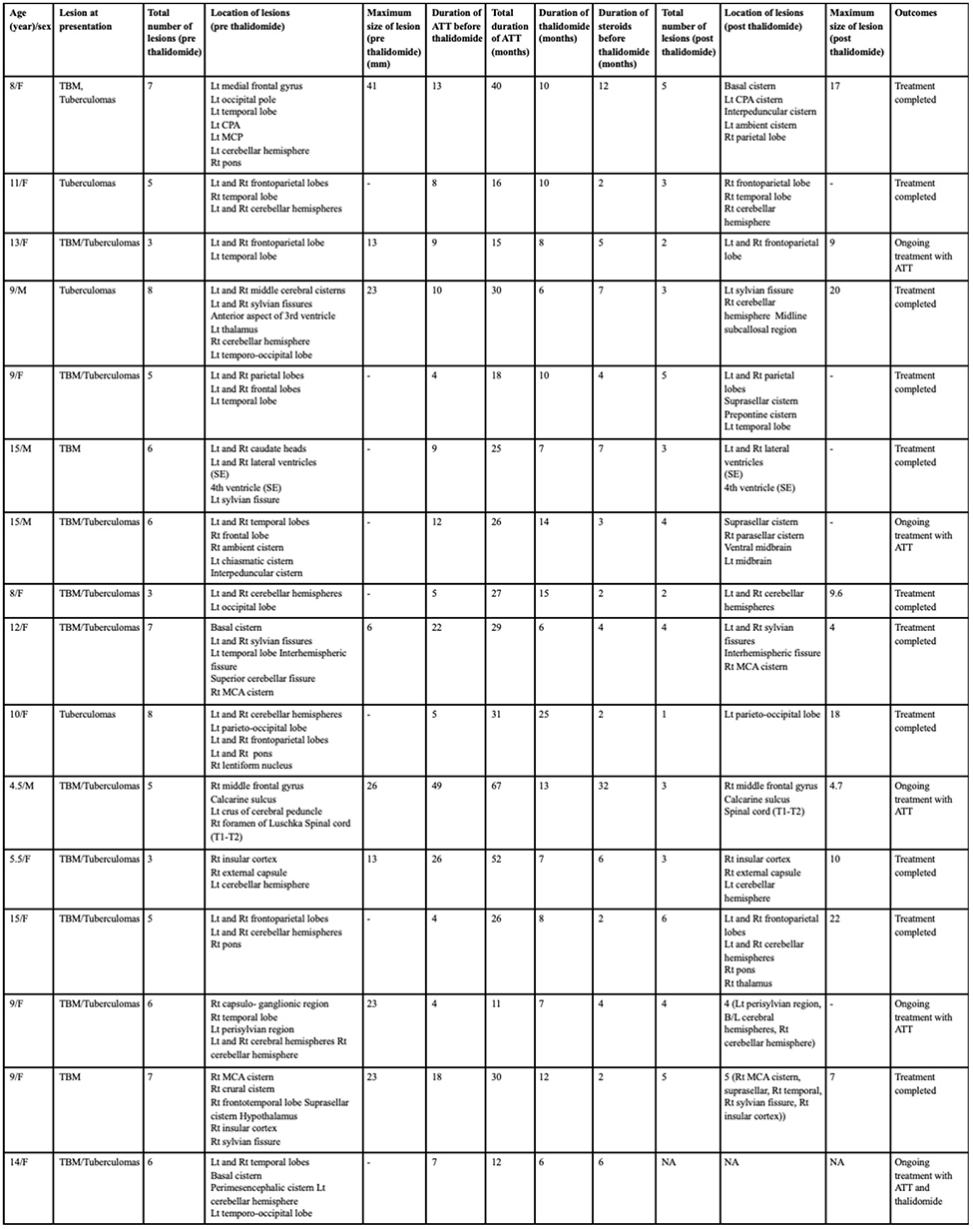
Individual patient characteristics including radiological, treatment and outcome details. Note: Lt- left, Rt- right, CPA- cerebellopontine angle, MCP- middle cerebellar peduncle, SE- subependymal, MCA- middle cerebral artery, NA- not available

**Results:**

The mean age at presentation was 10.38±3.37 years with a male-to-female ratio of 1:3. Thirteen (81.25%) patients had TB meningitis, and 14 (87.5%) had tuberculomas at presentation. The mean number of tuberculomas pre- and post-thalidomide was 5.6±1.68 and 3.53±1.36, respectively (p=0.001). The mean size of the largest tuberculoma pre- and post-thalidomide was 21±10.62mm and 12.13±6.55mm, respectively (p=0.044). Radiological improvement post-thalidomide was seen in 14 (93.33%) out of 15 patients. The mean duration of thalidomide received was 10.25±4.9 months, and the mean duration of ATT received was 29.06±14.91 months. Anemia was seen in 10 (62.5%) patients, hepatotoxicity in 4 (25%) patients, thrombocytopenia in 2 (12.5%) patients, and a skin rash in 1 (6.25%) patient.

**Conclusion:**

Thalidomide can significantly reduce the size and number of tuberculomas in pediatric CNS-TB patients, particularly in those who are steroid unresponsive or develop a paradoxical reaction to steroid and ATT.

**Disclosures:**

All Authors: No reported disclosures

